# Synthesis and Antimycobacterial Activity of 2-(Phenylthio) benzoylarylhydrazone Derivatives

**Published:** 2011

**Authors:** Ali Almasirad, Somayeh Samiee-Sadr, Abbas Shafiee

**Affiliations:** a *Department of Medicinal Chemistry, Facalty of Pharmacy, Pharmaceutical Sciences Branch, Islamic Azad University, Tehran, Iran.*; b *Department of Medicinal Chemistry, Faculty of Pharmacy and Pharmaceutical Sciences Research Center, Tehran, Iran.*

**Keywords:** Hydrazones, Antimycobacterial activity, Mycobacterium tuberculosis, 5-Nitrofuran, 5-Nitrothiophen

## Abstract

A series of new 2-(phenylthio) benzoylarylhydrazones were synthesized by acid-catalyzed condensation of hydrazide 3 with corresponding aldehydes. The chemical structures of the compounds were elucidated by FT-IR, ^1^H-NMR and Mass spectra. All newly synthesized compounds were evaluated for their antimycobacterial activities against *Mycobacterium tuberculosis *H_37_Rv using the microplatealamar blue assay (MABA). Compounds 4f **(**5-Nitro-2-furyl analogue) and 4g **(**5-Nitro-2-thienyl analogue) showed antimycobacterial activity with IC_90_, 7.57 and 2.96 μg/mL, respectively.

## Introduction

Tuberculosis (TB) is one of the first identified infectious diseases and remains a major health problem with two million deaths and eight million new cases annually (1). Resistance of *Mycobacterium tuberculosis *strains to antimycobacterial agents is an increasing problem world wide (2).

Excessive use of antibiotics is generally accepted to be the main reason for increased antibiotic resistance among bacteria (3-5). Over the past 50 years, no new drug classes have been introduced to the treatment of tuberculosis (6). Currently, patients require 6-9 months of treatment. This long period leads to the lack of compliance, which in turn, can be responsible for the relapse and emergence of resistant strains (7, 8). Thus, the developments of potent new antituberculosis drugs, which are active against resistant strains and latent forms and reduce the treatment period, are urgently needed to combat this disease.

The hydrazone type and nitroheteroaryl-containing compounds constitute important classes of biologically active drug molecules which have attractive attention of medicinal chemists due to their antituberculosis and antibacterial activity (9-13). However, there are some reports about the antitubercular activity of sulfur linked biaryl compounds (14, 15). In view of these data, we aimed at the synthesis and antituberculosis evaluations of new hydrazone derivatives.

## Experimental

*Chemistry*

The synthesis of hydrazone derivatives was performed following the steps shown in scheme 1. According to our previously described method, methyl 2-(phenylthio)benzoate 2 was prepared via esterification of 2-(Phenylthio)benzoic acid 1 and the key intermediate 2-(phenylthio)benzoic acid hydrazide 3 was prepared from the reaction of hydrazine hydrate with compound 2 (16). Target compounds were synthesized by acid-catalyzed condensation of hydrazide 3 with corresponding aldehydes (17, 18). The structures of the synthesized compounds were assigned on the basis of IR, ^1^H-NMR and Mass spectra.

Chemicals were purchased from Merck chemical company (Tehran, Iran). 5-Nitrothiophen-2-carboxaldehyde was purchased from Alfa Aesar Company. Melting points were taken of a Kofler hot-stage apparatus (Richert, Vienna, Austria) and are uncorrected. ^1^H-NMR spectra were obtained using a Bruker FT-80 spectrometer (Bruker, Rheinstetten, Germany). Mass spectra were obtained using a Finnigan-MAT TSQ-70 spectrometer at 70 eV (Finnigan, Bremen, Germany). The IR spectra were obtained using Nicolet FT-IR Magna 550 spectrographs (KBr disks) (Nicolet, Madison, WI, USA). Elemental analyses were carried out with a Perkin Elmer Model 240-c apparatus (Perkin Elmer, Norwalk, CT, USA).The results of the elemental analyses (C, H, N) were within ± 0.4% of the calculated amounts.

*General procedure for the synthesis of 2-(Phenylthio)benzoylarylhydrazones (4a-4h)*

A mixture of hydrazide 3 (4.1mmol) and corresponding aldehyde (4.3 mmol) in absolute ethanol (40 mL) was stirred at room temperature for 2 to 3 h in the presence of hydrochloric acid (2 drops) as a catalyst. The end of the reaction was observed with TLC and the hydrazones 4a-4h were isolated by concentration of the reaction mixture under reduced pressure, followed by neutralization with a 10% aqueous solution of sodium bicarbonate. The resulting precipitate was filtered, washed with water (20 mL) and crystallized from a suitable solvent.

*2-(Phenylthio)benzoic acid (Benzylidene) hydrazide (4a)*

Yield 71% , m.p 225-226°C (ethanol ); IR (KBr): 3206 (NH), 3047 (CH, aromatic), 1644 (C=O); ^1^H-NMR (CDCl_3_) 11.81 (bs, 1H, NH), 7.37-7.25 (m, 14H, aromatic); MS: m/z (%) 332 (M^+^,7), 213 (92), 181 (57), 92 (100), 63 (71); Anal. Calcd.for C_20_H_16_N_2_OS: C, 72.26; H, 4.85; N, 8.43. Found: C, 72.57; H, 4.76; N, 8.58.

*2-(Phenylthio)benzoic acid (4-Nitrobenzylidene) hydrazide (4b)*

Yield 95%, m.p 152-154°C (ethanol); IR (KBr): 3277 (NH), 3075 (CH, aromatic), 1664 (C=O), 1511, 1342 (NO2) ; ^1^H-NMR (CDCl_3_): 12.01 (bs, 1H, NH), 8.20 (d, J = 8.8Hz, 2H, aromatic), 8.14 (s, 1H, N=CH), 7.70-7.27 (m, 11H, aromatic); MS: m/z (%) 376 (m^+^-1, 23), 211 (100), 182 (80); Anal. Calcd.for C_20_H_15_N_3_O_3_S: C, 63.65; H, 4.01; N, 11.13. Found: C, 63.86; H, 3.94; N, 11.04.

*2-(Phenylthio)benzoic acid (4-Pyridinylidene) hydrazide (4c)*

Yield 86%, m.p 165-167°C (ethyl acetate); IR (KBr): 3277 (NH), 3047 (CH, aromatic), 1669 (C=O); ^1^H-NMR (DMSO-d6): 12.15 (bs, 1H, NH), 8.65 (bs, 2H, pyridine), 8.31 (s, 1H, N=CH), 7.61-7.19 (m, 11H, aromatic, pyridine). MS: m/z (%) 333 (M^+^, 14), 213 (64), 184 (37), 96 (86); Anal. Calcd, for C_19_H_15_N_3_OS: C, 68.45; H, 4.53; N, 12.6. Found: C, 68.57; H, 4.35; N, 12.68.

*2-(Phenylthio)benzoic acid (2-Furylidene) hydrazide (4d)*

Yield 88%, m.p 209-212°C (ethanol); IR (KBr): 3201 (NH), 3042 (CH, aromatic), 1639 (C=O); ^1^H–NMR (DMSO-d6): 11.7 (bs, 1H, NH), 8.20 (bs, 1H, N=CH), 7.84 -6.91 (m, 9H, aromatic, 2H, furan), 6.64 (bs, 1H, furan); MS: m/z (%) 322 (M^+^,10), 212 (100), 183 (72), 92 (20); Anal. Calcd.for C18H14N2O2S: C, 67.06; H, 4.38; N, 8.69. Found: C, 67.32; H, 4.29; N, 8.89.

*2-(Phenylthio)benzoic acid (2-Thienylidene) hydrazide (4e)*

Yield 97%, m.p 197-199°C (ethanol); IR (KBr): 3200 (NH), 3042 (CH, aromatic), 1639 (C=O); ^1^H-NMR (DMSO-d6): 11.61 (bs, 1H, NH), 8.25 (s, 1H, N=CH), 7.65-7.13 (m, 12H, aromatic, thiophene); Ms: m/z (%) 338 (M^+^, 14), 211 (100), 182 (86), 92 (58); Anal. Calcd.for C_18_H_14_N_2_OS_2_: C, 63.88; H, 4.17; N, 8.28. Found: C, 64.02; H, 4.33, N, 8.49.

*2-(Phenylthio) benzoic acid (5-Nitro-2-Furyliden) hydrazide (4f)*

Yield 84%, m.p 208-210°C (ethanol); IR (KBr): 3175 (NH), 3010, 2 970 (CH, aromatic, furan), 1649 (C=O), 1526, 1337 (NO_2_); ^1^H-NMR (DMSO-d6): 12.30 (bs, 1H, NH), 8.25 (s, 1H, N=CH), 7.71 (d, 1H, j = 4.1 Hz, 1H, furan), 7.55-7.02 (m,9H,aromatic,1H,furan). MS: m/z (%) 367 (M^+^, 28), 284 (6), 213 (100), 193 (12), 93 (14); Anal. Calcd.for C_18_H_13_N_3_O_4_S: C, 58.85; H, 3.57; N, 11.44. Found: C, 58.70; H, 3.68; N, 11.51.

*2-(Phenylthio) benzoic acid (5-Nitro-2-Thienyliden) hydrazide (4g)*

Yield 91% , m.p 178-180°C (ethanol); IR (KBr): 3180 (NH), 3010, 2975 (CH, aromatic, thiophene), 1649 (C=O), 1531, 1337 (NO_2_); ^1^H-NMR(DMSO-d6): 12.25 (bs, 1H, NH), 8.51 (s, 1H, =CH), 8.07 (s, 1H, thiophene), 7.61-7.13 (m, 9H, aromatic, 1H, thiophene).

MS: m/z (%) 383 (M^+^,14), 213 (100), 184 (11), 93 (7); Anal. Calcd.for C_18_H_13_N_3_O_3_S_2_: C, 56.38; H, 3.42; N, 10.96. Found: C, 56.29; H, 3.37; N, 11.12.

*Biological activity*

All of the compounds were screened at the TAACF screening (Tuberculosis Antimicrobial Acquisition and Coordinating Facility) by the US National Institute of health. Primary screening was conducted at a single concentration of 10 μg/mL against *Mycobacterium tuberculosis *H_37_Rv (ATTCC 27294), in BACTEC 12B medium, using the MicroplateAlamar Blue Assay (MABA) (19).

Compounds demonstrating at least 90% inhibition in the primary screening (IC_90_ ≤ 10 μg/mL) were retested at lower concentrations by serial dilution against *mycobacterium tuberculosis *H_37_Rv to determine the actual MIC, using the MABA method. The VERO cell cytotoxicity assay (50% inhibitory concentration IC_50_) was done in parallel with TB dose response assay to determine the selectivity index (SI), defined as the ratio of the measured IC_50_ (mammalian cell toxicity) to the IC_90_ (H_37_Rv) Mycobacterium tuberculosis.

## Results and Discussion

In this study, a new series of 2-(phenylthio) benzoylarylhydrazone derivatives were synthesized and evaluated against Mycobacterium tuberculosis. From eight tested compounds, two (4f and 4g) displayed significant inhibitory effects (IC_90 _≤ 10 μg/mL). Compound 4g showed an IC_90_ value of 2.68, IC_50_ value of 3.11 and the selectivity index (SI) of 1.05. Compound 4f showed IC_90_ value of 7.57, IC_50_ value of 2.92 and the selectivity index (SI) of 0.39 (Table 1). 

**Table 1:** Antitubercular activity screening data of the synthesized compounds

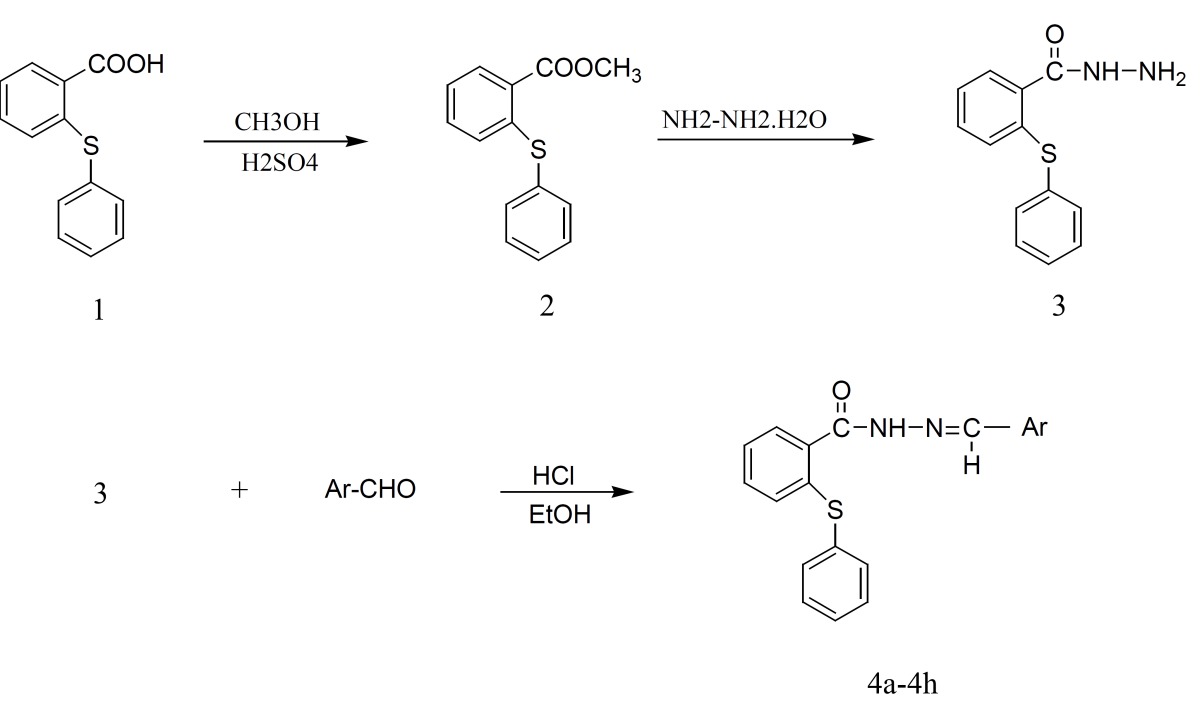

Despite the good activity of compounds 4f and 4g, their imidazole analogue, 4h, was weakly active. The results are in agreement with another research in which the comparison between nitroimidazole and nitrofuryl containing heteroaryl derivatives showed that the nitroimidazole ring resulted in compounds devoid of antimycobacterial activity (13). Similar to the previous study, the comparison of compounds (4b, 4f and 4g) with their inactive analogues (4a, 4d and 4e) showed that existence of nitro substitution on aryl part of the synthesized compounds can improve the activity (Table 1) (20). The results provide more evidence for the other study that showed the antituberculosis activity of nitrothiophen containing compounds (21).

These effective derivatives are ideally suited for further modification to obtain more active and less cytotoxic antimycobacterial compounds.

**Figure 1:**
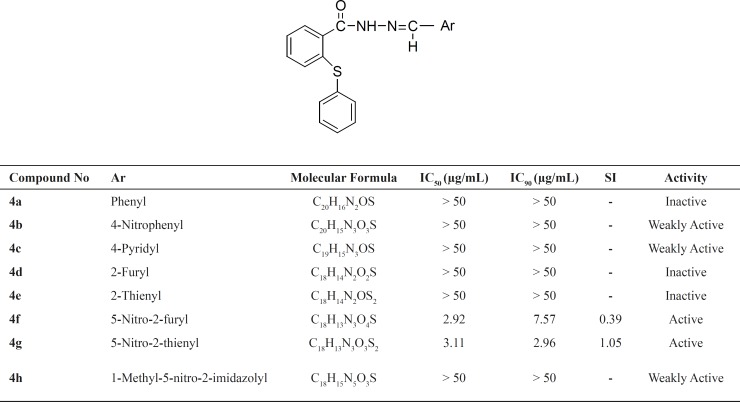
Synthesis of hydrazone derivatives
